# Acoustic Sensor Data Flow for Cultural Heritage Monitoring and Safeguarding

**DOI:** 10.3390/s19071629

**Published:** 2019-04-05

**Authors:** Panagiotis Kasnesis, Nicolaos-Alexandros Tatlas, Stelios A. Mitilineos, Charalampos Z. Patrikakis, Stelios M. Potirakis

**Affiliations:** Department of Electrical and Electronic Engineering, University of West Attica, 12244 Athens, Greece; ntatlas@uniwa.gr (N.-A.T.); smitil@uniwa.gr (S.A.M.); bpatr@uniwa.gr (C.Z.P.); spoti@uniwa.gr (S.M.P.)

**Keywords:** acoustic sensors, cultural heritage, sensor signal processing, sensor linked data, deep learning, ontologies, semantic rules, state of preservation monitoring

## Abstract

Cultural heritage sites, apart from being the tangible link to a country’s history and culture, actively contribute to the national economy, offering a foundation upon which cultural tourism can develop. This importance at the cultural and economic level, advocates for the need for preservation of cultural heritage sites for the future generations. To this end, advanced monitoring systems harnessing the power of sensors are deployed near the sites to collect data which can fuel systems and processes aimed at protection and preservation. In this paper we present the use of acoustic sensors for safeguarding cultural sites located in rural or urban areas, based on a novel data flow framework. We developed and deployed Wireless Acoustic Sensors Networks that record audio signals, which are transferred to a modular cloud platform to be processed using an efficient deep learning algorithm (f1-score: 0.838) to identify audio sources of interest for each site, taking into account the materials the assets are made of. The extracted information is presented exploiting the designed STORM Audio Signal ontology and then fused with spatiotemporal information using semantic rules. The results of this work give valuable insight to the cultural experts and are publicly available using the Linked Open Data format.

## 1. Introduction

Cultural Heritage (CH) is considered worldwide as part of peoples’ collective identity and strongly affects social cohesion and their sense of a common past and future. Moreover, exploitation of CH in the context of tourism has a high impact in European and global economies [1,2,3]. Therefore, the protection and preservation of CH should be a priority for our society. CH assets are extremely exposed to climate change, natural hazards and even anthropogenic risks, threatening their integrity and, probably, compromising their value. The loss or deterioration of these outstanding assets can negatively affect local and national communities, due to their cultural importance as a source of information on the past and a symbol of identity, as well as because of their socio-economic value. To this end, innovative environmental monitoring technologies and systems should be adopted to enable disaster prevention and quick damage assessment when catastrophes occur.

Acoustic sensors and their networks have proven their eligibility for scene analysis applications, and may represent a critical part of a heterogeneous sensors platform and related fusion techniques. This is reflected in directives of the European Union [4] as well as periodical reports of the US government [5]. An excellent example of the importance of acoustic sensors for scene analysis applications is the 9th annual MLSP competition that was specifically targeted to scene analysis and automatic recognition of bird species using audio samples collected in real-world field conditions [6]. Efficient sound classification can play an important role in CH safeguarding since it can be efficiently used in the assessment of human-originating actions, like vandalism or malevolent behavior, or natural phenomena with potential hazardous effects on cultural sites. In order to efficiently detect them, advanced Machine Learning (ML) techniques can be adopted, exploiting the potential of technological breakthroughs in Artificial Intelligence.

The rise of Deep Learning (DL), which is a branch of ML, has revolutionized the performance of data analysis in many computer tasks such as computer vision [7], speech recognition [8] and natural language processing [9], outperforming past techniques based on human-crafted features (HCFs). Its main advantage is that it has the ability of extracting features automatically [10,11,12], in contrast to conventional signal processing techniques that apply mathematical, statistical or heuristic functions to raw signals, in order to extract valuable HCFs.

However, for the CH experts who are interested in retrieving the situational pictures of hazardous events, extracted audio information is not useful if this is not further processed. In order to generate events from incoming sensor data, extracted information should be correlated with a specific space and time and eventually be presented using interactive plots. Thus, there is the need of developing a sensor platform capable of monitoring a CH site’s area and performing almost real-time data analysis.

Safeguarding Cultural Heritage through Technical and Organisational Resources Management (STORM), is a HORIZON 2020 funded European Union CH project [13]; it is a European research project that aims at the protection of CH through a combination of technical and organizational resources, including the development of Wireless Acoustic Sensor Networks (WASNs) for cultural sites monitoring. The integrated STORM sensors platform will secure sites’ monitoring and orchestrate stakeholders’ proactive actions in order to safeguard the preservation of CH. Moreover, STORM relies on an Internet of Everything platform [14] that is designed based on five use cases that highlighted the needs of five corresponding CH sites placed all over Europe (i.e., Great Britain, Italy, Greece, Portugal and Turkey). Several cutting-edge sensors were selected and implemented to be used for CH monitoring. Moreover, STORM takes advantage of the edge and cloud computing technologies to process and store the produced data and provide a cloud-based platform for stakeholders reporting and management information as well as reaction tools.

In this paper we present a data flow framework employed by the STORM platform and is based on acoustic sensors in order to enable the monitoring and safeguarding of CH assets. Starting from the collection of raw audio signals near the CH sites of interest, we describe in detail a data flow that is capable of accurately identifying the audio sources of the signals and a posteriori detect hazardous events to notify the CH stakeholders. In particular, the main contributions of this paper can be summarized in the following:

(1) Design and development of a Wireless Acoustic Sensor Network to record audio signals in remote areas
(2) Application of Transfer Learning to finetune a Deep Convolutional Neural Network in order to detect audio sources of interest
(3) Design of a flexible audio signal ontology that can be used to detect audio-based events for the Cultural Heritage domain
(4) Publication (in the publicly available STORM cloud) of the generated audio-based events exploiting the Linked Open Data paradigm.

The rest of the paper is organized as follows: in the next section we survey the recent developments in the field of audio signal recording, processing, and applying semantic information fusion. In Section 3 we describe in detail the proposed event detection methodology. In particular, we illustrate the developed acoustic sensors, the architecture of the DL model that is used to process audio signals, and the ontologies that were designed to develop the semantic rules. In Section 4 we demonstrate the obtained results of the DL algorithm and the rules that are utilized to detect events and publish them over the network. Finally, Section 5 concludes this work and proposes future steps.

## 2. Related Work

### 2.1. Wireless Sensor Networks

**Figure 1:**
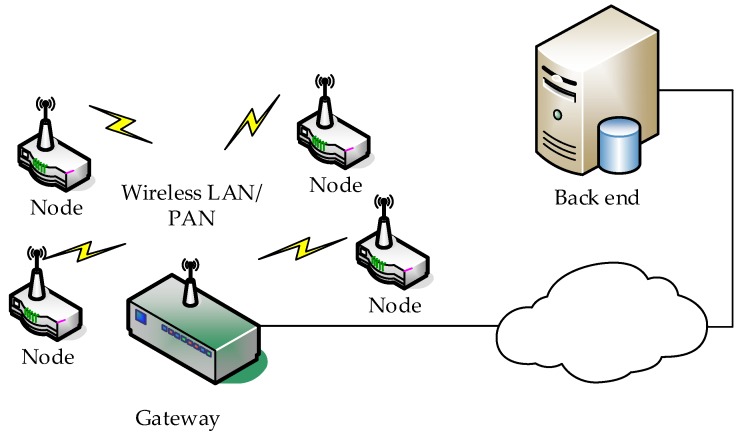
WSN architecture.

A Wireless Sensor Network (WSN) is a network based on devices, namely sensor nodes, aiming to monitor one or multiple physical/chemical quantities. These nodes are usually dispersed over an area and communicate via a wireless link in order to exchange information and ultimately consolidate the data to a single node/gateway for further processing and possibly for transmission to a back end [15]. Figure 1 shows a general WSN architecture, including the entities defined above.

WSNs provide a superior solution compared to traditional setups, presenting numerous advantages such as lower installation and operation cost, adaptability to requirements, resilience, and virtually infinite spatial coverage. The main challenges for the broad adoption of WSNs are their power requirements, as most applications are off-grid, as well as the supported range and maximum bitrate of Wireless protocols. Power harvesting of renewable sources [16], such as vibration, solar and wind energy, and newer networking systems including base station based links [17] offer potentially viable solutions to these challenges. WSNs may be employed in a wide variety of applications such as environmental [18], defense/security [19] and welfare [20].

A Wireless Acoustic Sensor Network (WASN) is a WSN where the physical quantity observed is an audio signal captured through a microphone. Since the data rate of any audio signal, even for speech-quality recordings, is orders of magnitude higher compared to that of signals usually handled by WSNs, increased node processing power is necessary, beginning with mandatory preconditioning and reaching to compression or full-scale analysis, WASNs are considered to be a separate WSN category. Example applications of WASNs are noise monitoring [21], sound classification [22] as in road traffic, horns, and people, ambient assisted living [23], security [15] for explosion/gunshot detection and localization, as well as environmental monitoring [24].

A WASN may transmit the actual audio signal, possibly preprocessed to improve the useful part, and/or features extracted from the data that may be used in order to categorize the captured sound via classification [25]. Audio transmission raises the need for an increased bitrate: depending on each node duty cycle, this may approach audio streaming. Real time signal compression [26] is another viable option, which in turn increases the node computational effort. Alternatively, feature extraction, depending on the target application, may range from straightforward operations such as band filtering and Sound Pressure Level calculation to complex mathematical features such as the estimation of complexity measures [27]. The advantage of audio transmission / back-end archiving is that the data may be re-processed in the future using emerging algorithms while human annotation and accuracy estimation may be performed; feature transmission on the other hand has far less bitrate and data storage requirements.

Based on the trade-offs between the wireless protocol maximum bitrate the node processing capabilities and power consumption, the node cost and area to be monitored, specific design decisions usually lead to a unique WASN for each application.

### 2.2. Audio Signal Processing

Audio signal processing relies on carefully selected sound attributes that correspond to specific signal characteristics (i.e., sound features that are then fed to an appropriate classifier). A variety of sound features have been proposed for efficient scene analysis and monitoring, and they generally fall in either of two main categories: time-domain or frequency-domain features. Example of the former include the zero-crossing rate (ZCR), linear prediction coefficients (LPC), audio signal energy function, volume, etc., while examples of the latter include the pitch, bandwidth, fundamental frequency, spectral peak track, brightness, and so forth. On the other hand, statistical features include the variance, skewness, kurtosis, median, mean value, as well as various complexity measures (entropies, information) of the signal [27,28,29]. Other spectral features used in the literature include the 4-Hz modulation energy, percentage of low frames, spectral centroid, spectral roll-off point, spectral frequency, mean frequency, and high and low energy slopes [30,31,32].

Nonetheless, time- or frequency-domain or statistical features are often extracted from the whole signal and so don’t include information about its temporal evolution. However, temporal evolution may reveal important aspects of the underlying events that are sometimes critical regarding their successful classification. Therefore, single-value features alone are not enough to represent environmental audio signals that are highly non-stationary in nature. In order to overcome this problem, time-frequency (TF) features have been introduced in order to capture the temporal variation of the spectra of such signals. TF features are essentially time- or frequency-domain features that are calculated in small frames of the entire sound signal; the resulting frame-indexed features are thus forming a time-indexed sequence. More often than note, adjacent signal frames are overlapping with each other in order to smooth out the resulting TF sequence. TF features include the spectrograms, scalograms, mel-frequency cepstral coefficients (MFCC), short Fourier coefficients, and other.

TF features may be fed to classifiers like Hidden-Markov Models (HMMs) but also to artificial neural systems, like in this paper [33]. In our case, the dimensionality of the input space of TF features makes it prohibitive to feed them directly to a classifier operating at the WASN edge nodes. Thereupon, data dimensionality reduction needs to precede the feeding of the classifier. In this respect, different solutions for the reduction of data dimensionality have been proposed; an effective proposed solution that is also used herein is to use Gaussian Mixture Models (GMMs) [34]. GMMs are used in order to estimate the probability distribution function of time-frequency features over all frames and feed the underlying classifier with the estimated parameters of the GMM, thus providing an input of significantly reduced size.

On another page, sound classifiers broadly fall into two categories: discriminative and non-discriminative. Examples of the former include the k-means classifier, the polynomial classifier, the multi-layer perceptron (neural network) and the support vector machines, while examples of the latter include the HMM, which attempt to model the underlying distribution of the training data [35,36,37]. For the proposed classification platform of the STORM project we selected to use a generic discriminative classifier, i.e., an Artificial Neural System (ANS), and more specifically a deep convolutional implementation, since deep networks are well-known classifiers that have been extensively used for signal and audio classification purposes (see following sub-sections). Finally, it is worthwhile noting that wavelet analysis has been also used as signal pre-processing in order to partially remove ambient noise [37].

### 2.3. Deep Learning on Audio Signals

Similar to the task of computer vision, Convolutional Neural Networks (CNNs or ConvNets) are the state-of-the-art DL algorithm for audio classification [38]. The main advantage of a convolution operation (Equation (1)) is that it manages to obtain a less noisy estimate of a sensor’s measurements by applying filters on them and average their output afterwards. For example, if some measurements are more accurate and some are not (e.g., due to noise), the accurate ones should contribute more to the average, thus, the sensor’s measurements are convolved with a weighting function *w* [39]. 

ConvNets are designed to process data that have temporal local dependencies, such as images and sensor signals. Consequently, in the case of audio signals, the input, which is the preprocessed and segmented audio signal, is converted to a log-scaled mel-spectrogram and is combined with the 2D filters (weights). These filters are trained (updated) in order to discover the most suitable patterns (e.g., peaks in the signal). The output of the convolution is called the activation map, which is the product of several parallel convolutions between the signal and the filters.

The *i-*th, *j-*th product element of a discrete 2D convolution between input matrix *x* and 2D filter *w* equals:

(1) $$c_{i,j}^{l,q}=b^{l,q}+\sum_{h=1}^{H}\sum_{d=1}^{D}w_{d,h}^{l,q}x_{i+d-1,i+h-1}^{l-1,q}$$

where *l* is the layer index, *q* is the activation map index, *D* is the total width of the filter *w*, b is the bias term and *h* is the total height of the filter *w*.

**Figure 2:**
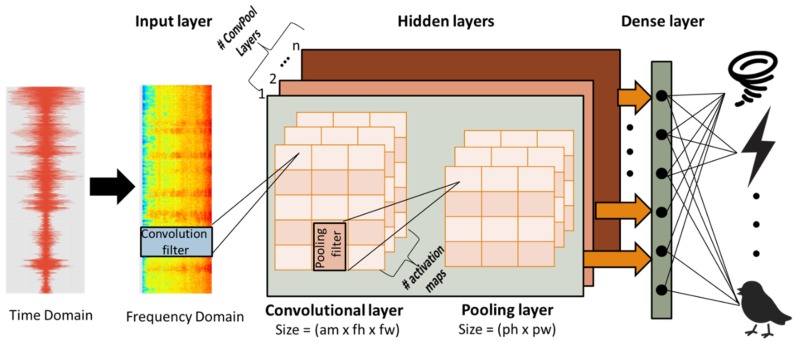
Audio classification based on ConvNets.

The output of the convolutional layers that detect local conjunctions of features from the previous layer, is passed to a non-linear activation function, such as Rectified Linear Unit (ReLU) [7], which thresholds all values at zero. Afterwards, the output values are fed to the pooling layers that merge semantically similar features into one [10]. A typical pooling unit computes the maximum or the average of a local patch of units in one or more activation maps. Two or three stages of convolution layers, non-linear activation functions and pooling layers are stacked, and are followed by fully-connected layers, and a *softmax* activation function to estimate the probability of a certain class *k*. Finally, using a loss function we estimate how accurate the ConvNet is and by applying backpropagation we compute the weight gradients in order to update the convolutional filters of the deep network, and eventually train the model. Figure 2 illustrates a deep ConvNet used to predict the source of an audio signal.

One of the main advantages of DL algorithms, such as Deep CNNs, is their ability to increase their performance as the training dataset grows. Hersey et al. [38] used well-known CNN Architectures, which have been applied successfully in computer vision tasks, to test their effectiveness on classifying large-scale audio data. In particular, they applied a baseline fully connected ANN, an AlexNet [7], a VGG [40], an Inception V3 [41], and a ResNet-50 [42] to the AudioSet [43], which consists of 2,084,320 human-labeled 10-second audio clips. The ResNet-50 model, which had the most layers (i.e., it was deeper than the others), achieved the best results.

CNNs are also state-of-the-art even for relatively smaller audio datasets, consisting of 1 to 10 thousand samples. Thus, based on the UrbanSound8K [44], Salamon and Bello [45] compare a baseline system (i.e., using MFCCs features) with unsupervised feature learning performed on patches of PCA-whitened log-scaled mel-spectrograms. In particular, they used the spherical k-means algorithm [46] followed by the Random Forests algorithm and they managed an average classification accuracy 5% higher than the baseline system. A few months later, Piczak [47] achieved to obtain state-of-the-art results, based on a relatively shallow CNN (2 convolutional layers), having as input log-scaled mel-spectograms of the clips provided by the UrbanSound8K dataset. Even though the proposed CNN model seemed to overfit the training data, its average accuracy was about 73.1%, against the 68% average accuracy of the baseline model. Moreover, Kumar [48] used a deeper, Vgg-like, CNN model (five convolutional layers) on the UltraSound8K dataset and reached a 73.7% average accuracy.

Furthermore, in order to increase the size of the datasets, data augmentation techniques have been adopted by researchers. Piczak [49] utilized random time delays to the original recordings of the ESC-50 (2000 clips) and ESC-10 (400 clips) datasets. In both cases, his CNN architecture achieved better accuracy results than the baseline model, and in the case of the ESC-50 the difference between the average accuracies was above 20% (baseline accuracy: 44%, best CNN: 64.5%). In addition, Salamon and Bello [50] explored the influence of different augmentations on the performance of a proposed CNN architecture and obtained an average accuracy close to 79%. The data augmentation techniques they used were: (a) Time Stretching, (b) Pitch Shifting, (c) Dynamic Range Compression and (d) Adding Background Noise.

Kumar et al. [51] applied Transfer Learning (TL) from weakly labelled audio using CNN for sound events and scenes. TL is based on the intuition to transfer the extracted features (keep the weights fixed) of a trained model on a specific dataset to another dataset (usually smaller). In particular, they trained a VGG-like using the AudioSet, a dataset considered to be weakly labelled since in most of the clips the sound event (e.g., thunder) is assumed to be present in the whole audio recording, while it is not. The authors used the extracted features to create another model in order to be applied to the ESC-50 dataset, the networks accuracy achieved to overcome even the performance of a human being (83.5% to 81.3% [49]).

Finally, a semi-supervised TL approach was adopted in [52], where the authors designed a system that is able to learn both visual and audio semantic information in a completely unsupervised manner by simply looking at and listening to a large number of unlabeled videos, using AudioSet as training dataset. In particular, they used a deep ConvNet that learns to extract features by determining whether a pair of (video frame, short audio clip) correspond to each other or not. Afterwards, they applied TL, keeping only the audio related features, using the ESC-50, and achieved 79.3% accuracy.

### 2.4. Semantic Reasoning for Audio-Based Event Detection

Complex Event Processing (CEP) is a technique that is used in the domain of CH information aggregation and correlation to identify potentially dangerous or critical situations that may suggest the need for monitoring, surveying or warning for disaster prevention and risk assessment [53]. A well-investigated technique for fusing audio information is that of exploiting Sematic Web technologies, such as ontologies and semantic rules.

By the term ontology, we denote a formal explicit specification of a shared conceptualization where, conceptualization is an abstract, simplified view of the world for describing semantically the objects, concepts and other entities, existing in a domain along with their relationships [54]. Most of the existing audio ontologies cannot be used for event detection, since they mainly focus on representing the audio features of a signal [55,56], such as amplitude and audio pitch, to be applied to the music domain, or they can be considered more as a taxonomy [57]. Semantic rules are used to apply deductive reasoning to data expressed by an ontological schema, and consist of a set of positive conjunctions of atoms [58]. As defined in propositional logic, a propositional sentence is valid if all the atoms of the antecedent part, also called as body, of the sentence are true. Therefore, if the body of a rule is valid then the consequent, also called as head, is activated. A semantic rule is expressed as:

p(arg1,arg2,…,argn) ^ p(arg1,arg2,…,argn) ^ … ^ p(arg1,arg2,…,argn) → p(arg1,arg2,…,argn)(2)

where the term p(arg1,arg2,…,argn) denotes an atom. The p character is the predicate symbol and the arg1,arg2,...,argn are the arguments of the expression. The p symbol may represent classes, object properties or datatype properties. The arguments can be individuals or data values, or variables denoting them.

A system of ontology-based sound retrieval is proposed by Hatala et al. in [59] to serve museum visitors. The ontology they introduce is used to describe concepts and characteristics of sound objects as an interface between users and audio database, while the retrieval mechanism is based on semantic rules. The audio ontology presented in [60] focus on the representation of audio sources in the domain of telemedicine (e.g., detect situations of distress in a patient’s room based on sound analysis). Finally, a reasoning framework, which combines the audio-visual cues with violence and multimedia ontologies to detect the type of violence (e.g., fight, screams, gunshots), is introduced in [61]. 

## 3. Acoustic Data Flow on the STORM Platform

The STORM platform follows a Service Oriented Architecture (SOA) to achieve an efficient data flow, while the services are composed in a layered style, offering benefits in terms of interoperability, understandability and reuse. In particular, the architectural layers communicate with each other by exposing an interface (i.e., API). In this section, we focus on explaining in detail the data flow that is initiated by the acoustic sensors, however we firstly describe the modular architecture of the STORM platform.

As aforementioned, STORM platform is based on the IoE paradigm, providing the data produced by IoT devices (i.e., weather, environmental and structural monitoring sensors) to be processed by software defined services (e.g., audio classification) and to human experts. However, most of the deployed sensors produce a huge volume of data that has to be transmitted over the network to the cloud infrastructure in order to extract useful information. Thus, we have selected a tree-based cloud architecture; it consists of one Core cloud and several Edge clouds. The Edge clouds are cloud environments located near the sites of interest. Edges cloud are responsible for collecting, storing and processing the incoming data. In order to achieve low latency, regarding data transfer, in network-constrained environments (e.g., Roman Ruins of Tróia) Edge clouds should be located near each STORM site.

The Core cloud is responsible for the collection of extracted information from the Edge clouds, the generation of events by correlating information. Moreover, it monitors the status of the Edge clouds, and their services running on virtual instances. Since it is aware of which type of data each Edge cloud offers, it may interact with the Edge clouds to retrieve data and information related to the existing sensors in order to feed interactive visualization services. This type of communication between the Core cloud and the Edge clouds is accomplished with the use of RESTful APIs and the Publish/Subscribe (Pub/Sub) pattern.

**Figure 3:**
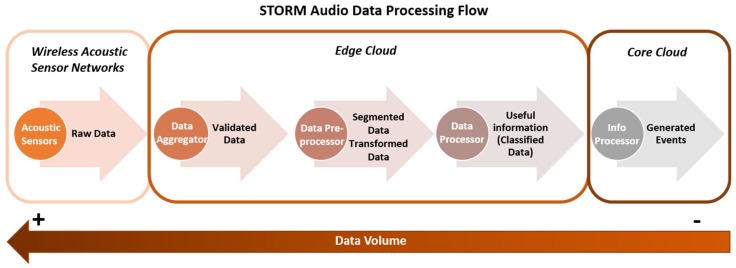
Audio Data Processing flow.

Figure 3 illustrates the data processing flow that takes place on the STORM platform regarding the collected audio signals. Initially, the audio signals are produced by the Wireless Acoustic Sensor Networks (WASNs) that are deployed near the sites of interest and are emitted to Edge cloud. In particular, the Data Aggregator component receives and validates them (i.e., check for missing values), forwarding them, afterwards, to the Data Preprocessor component. There, the signals are segmented into samples and transformed into the frequency domain in order to feed the machine learning classifier (i.e., Data Processor), which is responsible for extracting useful information from the audio samples by recognizing the source that produced it (e.g., sea waves). Finally, the extracted information is sent to Information Processor that is a software component hosted in the Core cloud and is responsible for detecting possible hazardous events. It should be noted, that during the data processing flow the volume of the data is reduced.

### 3.1. Wireless Acoustic Sensor Networks

The WASN developed and employed follows the general WSN architecture presented in Figure 1. Specifically, a number of nodes are installed in order to spatially cover the defined area and monitor for sound information. These nodes record and preprocess audio signals and are wirelessly connected to a node acting as a gateway; the gateway is responsible for packing and transmitting the data to the back end. 

**Figure 4:**
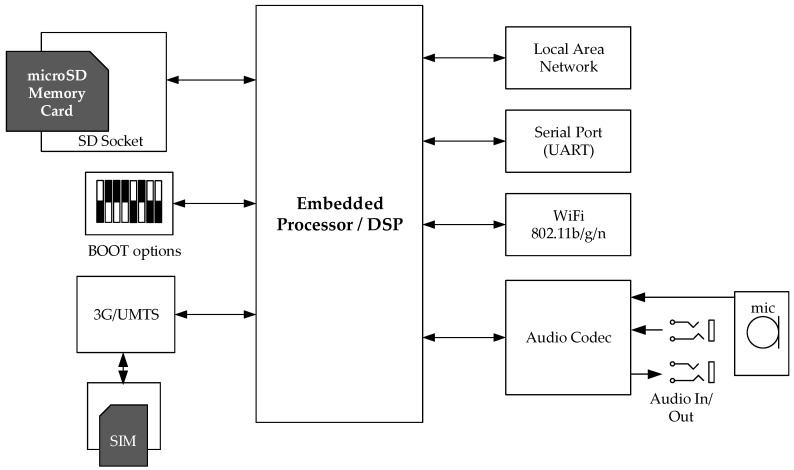
Hardware architecture of the WASN node.

The node architecture is shown in Figure 4. Each node was designed based on an ARM embedded processor/digital signal processor (DSP) [62], to ensure adequate data handling and local signal processing capabilities for audio streams. Specifically, the audio subsystem includes an audio codec that may facilitate the connection of analogue and digital microphones as well as analogue line-level inputs and outputs. One high-fidelity digital microphone [63] is used in our system, while the analogue inputs and outputs are used for debugging and testing purposes. The sampling frequency and bit resolution were chosen to be 48 kHz and 24 bits respectively (effectively 18 bits as per microphone specifications) to achieve the highest fidelity audio recording possible. A thresholding function has been implemented on the node, so (a) no audio is captured for signals below a predetermined sound pressure level and that (b) recordings are performed for signals above the threshold. The audio data are then pre-processed (e.g., bitwise operations may be necessary) and formatted (header introduction and segmenting) for transmission. While this setup favors an increased bandwidth requirement over local processing, audio data integrity is ensured, as no dropped frames or other distortions due to lack of local resources have been noted. The computationally-demanding recognition tasks are executed at a back-end server.

The communication peripherals on the node include (a) UART/Serial port used for software development and debugging. (b) WiFi [64] for node-to-gateway audio transmission, supporting multiple audio streams, (c) Local Area Networking-Ethernet and (d) mobile 3G/UMTS [65] data connection. The two latter are employed depending on availability in order to forward the information captured to the back-end for further processing. The firmware/software is stored and executed from an SD memory card, which is also used to temporarily save and que audio data in case of network downtime or insufficient bandwidth. The software employs the DSP for the acquisition, the pre-processing and formatting, while the ARM core is responsible for the system regulation and all extra-node messaging.

### 3.2. Deep Learning Model Architecture

In order to develop an accurate DL model, we reproduced the network introduced in [51]. In particular, the authors have uploaded the weights of the model they trained using AudioSet [66]. Moreover, we converted the audio signals into log-scaled mel-spectrograms using 128 mel bands, a window size of 23 ms and an overlap of 11.5 ms. Log-scaled mel-spectrograms have proven to be an suitable and well-investigated choice to be used as input for deep CNNs [38,48,49,51,52].

**Figure 5:**
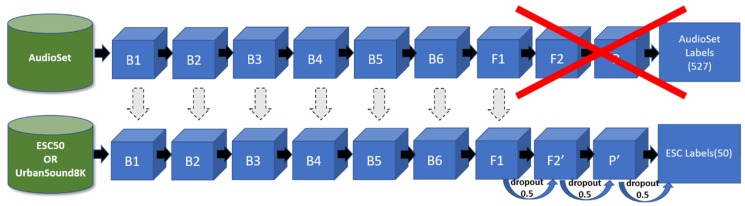
The proposed deep CNN architecture for the Audio Classifier component.

The network architecture (Figure 5) consists of several consecutive blocks, denoted with *B*, followed by two convolutional layers, represented by *F* and a global average pooling layer, represented by *P*. The exact architecture used in [51] is as follows:

- *B1*: 16 convolutional filters with a size of (3, 3), i.e., *W*^1^ has shape (3, 3, 1, 16). This is followed by batch normalization and a ReLU activation function. The output is convolved with another 16 convolutional filters with a size of (3, 3), i.e., *W* ^2^ has shape (3, 3, 16, 16). This is followed by batch normalization and a ReLU activation function, and a (2, 2) strided max-pooling operation.
- *B2*: 32 convolutional filters with a size of (3, 3), i.e., *W*^3^ has shape (3, 3, 16, 32). This is followed by batch normalization and a ReLU activation function. The output is convolved with another 32 convolutional filters with a size of (3, 3), i.e., *W* ^4^ has shape (3, 3, 32, 32). This is followed by batch normalization and a ReLU activation function, and a (2, 2) strided max-pooling operation.
- *B3*: 64 convolutional filters with a size of (3, 3), i.e., *W*^5^ has shape (3, 3, 32, 64). This is followed by batch normalization and a ReLU activation function. The output is convolved with another 64 convolutional filters with a size of (3, 3), i.e., *W* ^6^ has shape (3, 3, 64, 64). This is followed by batch normalization and a ReLU activation function, and a (2, 2) strided max-pooling operation.
- *B4*: 128 convolutional filters with a size of (3, 3), i.e., *W*^7^ has shape (3, 3, 64, 128). This is followed by batch normalization and a ReLU activation function. The output is convolved with another 128 convolutional filters with a size of (3,3), i.e., *W* ^8^ has shape (3, 3, 128, 128). This is followed by batch normalization and a ReLU activation function, and a (2, 2) strided max-pooling operation.
- *B5*: 256 convolutional filters with a size of (3, 3), i.e., *W*^9^ has shape (3, 3, 128, 256). This is followed by batch normalization and a ReLU activation function. The output is convolved with another 256 convolutional filters with a size of (3, 3), i.e., *W* ^10^ has shape (3, 3, 256, 256). This is followed by batch normalization and a ReLU activation function, and a (2, 2) strided max-pooling operation.
- *B6*: 512 convolutional filters with a size of (3, 3), i.e., *W*^11^ has shape (3, 3, 256, 512). This is followed by batch normalization and a ReLU activation function, and a (2, 2) strided max-pooling operation.
- *F1*: 1024 convolutional filters with a size of (3, 3), i.e., *W*^12^ has shape (3, 3, 512, 1024). This is followed by batch normalization and a ReLU activation function.
- *F2*: 527 convolutional filters with a size of (3, 3), i.e., *W*^13^ has shape (3, 3, 1024, 527). This is followed by batch normalization and a sigmoid activation function.
- *P*: a global average pooling function that has as input a tensor of shape (527, 10, 1) and as output a matrix of shape (527, 1). As a result, it averages the segments (~1 sec).
- *output*: 527 hidden units, i.e., W has the shape (527, 527), followed by a softmax activation function.

In order to apply Transfer Learning, we used the trained weights from *B1* to *F1*, and replaced the final layers and with two new layers *F2’, P’* (Figure 5):

- *F2’*: 256 convolutional filters with a size of (3, 3), i.e., *W*^13^ has shape (3, 3, 1024, 256). This is followed by batch normalization and a sigmoid activation function.
- *P’*: a global max pooling function that has as input a tensor of shape (256, number of segments, 1) and as output a matrix of shape (256, 1).

The output’s layers hidden units equal to the number of target classes. Thus, in case of the ESC-50 dataset they are 50. In both cases a *softmax* activation function was used to calculate the output probabilities.

### 3.3. STORM Audio Ontology for Event Generation

STORM Audio Signal Ontology (SAS-Ont) [67] describes the properties of an audio signal that are useful for the audio-based identification of hazardous events regarding the protection of CH. Such events can be related to humans (e.g., vandalism), machines (e.g., sound pollution), environmental phenomena (e.g., sea waves) or animals (e.g., snakes hatching). After the preprocessing phase the signals are fed to the deep ConvNet algorithm that outputs the source of the audio signal (e.g., thunder). Ontologies are an appropriate way of representing the extracted knowledge and via SPARQL queries and semantic rules more information can be inferred. For example, human voices recorded near a site during the day may not trigger an alert, but if they were recorded when the monument is closed for the visitors it may mean that a hazardous situation is taking place near the site.

**Figure 6:**
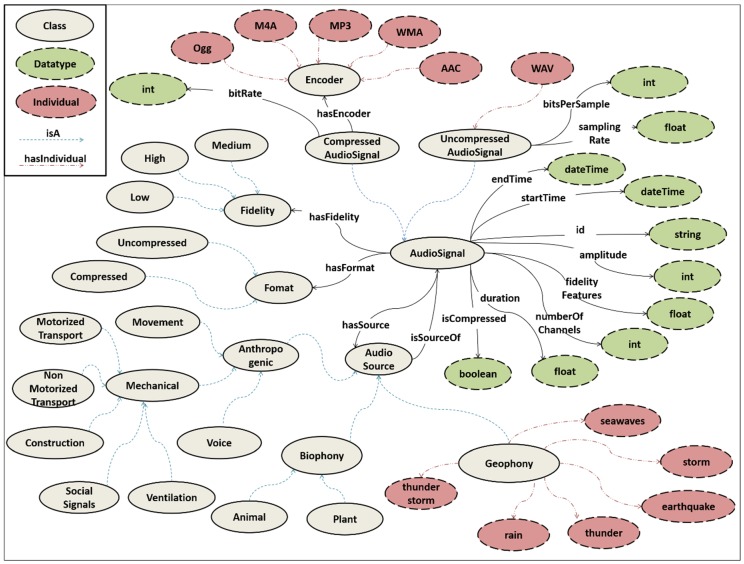
STORM Audio Signal Ontology.

Figure 6 presents the main classes of SAS-Ont, which is based on the taxonomy presented in [68]. The *AudioSignal* denotes the audio file that has been recorded and segmented. The *startTime* and *endTime* datatype properties identify the starting and the ending time respectively, while its duration is given by the *duration* datatype property. What is more, *id* is the audio signal’s identifier, *maxAmplitude* describes the intensity of the signal, *numberOfChannels* the channels of the signal and its *Fidelity* is given by a set of *fidelityFeatures*. The *Fidelity* class may be equal to *Low*, *High* or *Medium*. Based on the *Format* class, *AudioSignal* is further classified into *CompressedAudioSignal*, which means that the *isCompressed* property has value true and *UncompressedAudioSignal,* each one having each own properties. The *hasEncoder* object property links the *CompressedAudioSignal* with an *Encoder* (*AAC*, *MP3*, *M4A, Ogg*, or *WMA*). 

Finally, the *AudioSource* class has as main subclasses: (a) *Anthropogenic*, (b) *Biophony* and (c) *Geophony*. The *Anthropogenic* class is further classified into *Mechanical* sounds (e.g., *Car*) and sounds produced directly by a human being (e.g., *laughter*), while *Biophony* contains sounds produced by animals or plants. On the other hand, *Geophony* consists of sound categories related to natural hazards for the CH assets (e.g., *seawaves*). It should be noted that *AudioSource* points to *AudioSignal* via the *isSourceOf* property, while the audio signals are assigned to a source after been identified using a machine learning classification algorithm.

**Figure 7:**
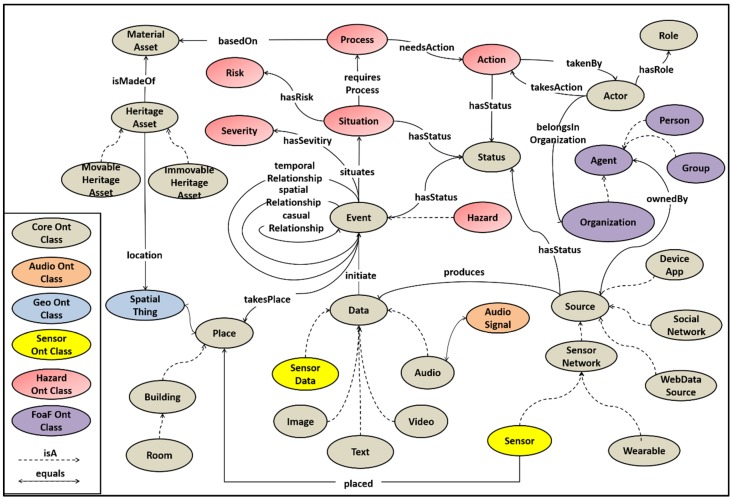
STORM core and domain ontologies.

SAS-ont is part of the STORM core ontology. Figure 7 represents the object relationships of STORM core ontology [69] and the links with the other STORM domain ontologies (i.e., STORM Sensor ontology [70], SAS-ont, and Event ontology [71]), FOAF [72], and Geo [73]. In particular, the *HeritageAsset* class represents the monuments and has as subclasses the *MovableHeritageAsset* and the *ImmovableHeritageAsset* entities. The *isMadeOf* property links the monuments with the *MaterialAsset* class, while the location property denotes the place the geographical point (*SpatialThing* or *Place*) of their location. *SpatialThing* is the main class of the Geo RDF vocabulary that provides the Semantic Web community with a namespace for representing latitude, longitude, altitude and other information about spatially-located things. The *Event* class is considered to be the most important class of our model, since it triggers the Quick Damage assessment services and select the best actions to deal with the emergency considering the known risks. 

Moreover, events may be linked with each other based on casual, temporal or spatial relationships. Events are initiated by the *Data* that the STORM platform collects. *Data* (*SensorData*, *AudioData*, *Image*, *Text*, and *Video*) are produced by a *Source* (*SensorNetwork*, *SocialNetwork*, *DeviceApp* or *WebDataSource*) that is *ownedBy* an *Agent* (i.e., an *Organization*, a *Person*, or a *Group*). The *Agent* class and its subclasses are entities of FOAF, which is a vocabulary used to link people and information using the Web. In addition to this, an *Event* has a *Severity* and may originate a *Situation*. If the *Situation* is hazardous it *requiresProcess* some *Process*, which is *basedOn* the *MaterialAsset* and needs an *Action*. An *Action* is *takenBy* an *Actor* that may belong to an *Organization*.

## 4. Results and Discussion

### 4.1. Experimental Sites

Three pilot sites of the STORM project have been identified that need to monitor their surroundings utilizing acoustic sensors, in order to attain the best management of maintenance, prevention, mitigation and response to severe impacts of potentially hazardous threats. In particular, the STORM pilot sites that identified the need of acoustic monitoring were:

- Baths of Diocletian (BOD),
- Roman Ruins of Tróia (RRT), and
- Mellor Archaeological Trust (MAT).

The main differences between the audio sources per site of interest may be summarized as:

- RRT site is located on a peninsula in Portugal, and as a result its stakeholders need to be aware of large sea waves,
- The MAT site is located on the edge of Stockport in Greater Manchester, which is a rural area and its stakeholders need to be aware of the potential for vandalism events, and
- BOD site, in contrast with the other two sites, is located in a crowded area (in the city of Rome), near the main train station. As a result, anthropogenic sounds are very common (i.e., not classified as abnormal activity), even in the middle of the night, and do not necessarily raise any suspicion.

Based on the description of the audio-based requirements and on the capabilities of the Audio Classifier component, we created a list of Audio Sources of Interest (ASoIs) that our service should identify almost in real-time, in order to notify the stakeholders of each site on time. Table 1 illustrates the ASoIs regarding the three sites that we have deployed WASNs.

**Figure 8:**
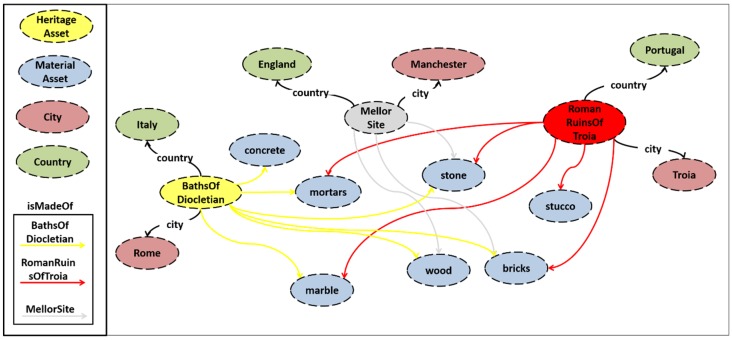
The ABox of the STORM Core Ontology regarding the material assets.

As a result, each CH site has its own specific features and its monitoring needs are based on the materials that its assets are made of. Figure 8 presents the relationships between the individuals (ABox) of the core ontology. An ABox is a set of facts (instances), while a TBox is the knowledge base. The *BathsOfDiocletian* monument is located in Rome, Italy. It *isMadeOf*: *concrete, mortars, stone, bricks, marble,* and *wood*. The *RomanRuinsOfTroia* monument is placed in Tróia of Portugal and *isMadeOf*: *stucco, mortars, stone, bricks, marble,* and *wood.* Finally, *stone, wood* and *bricks* are the *MaterialAssets* that were used to build the *MellorSite*, in Manchester.

### 4.2. Datasets and Experimental Results of Deep ConvNet

In order to test the proposed machine learning model and apply transfer learning, we used the extracted features provided in [66] to be used in the ESC-50 dataset; it is a freely available collection of short environmental recordings presented in a unified format (5 second-long clips, 44,100 Hz, single channel, Ogg Vorbis compressed at 192 kbit/s). All the available audio clips have been extracted and manually labelled by the author from public field recordings available through the Freesound project [74]. The dataset consists of a labelled set of 2000 environmental recordings (50 classes, 40 clips per class). The main reason for selecting this particular dataset is that it contains all the ASoIs for the STORM project.

The experiments were executed on two computer workstations in order to accelerate the training process. The first one is equipped with a NVIDIA GTX Titan X GPU, featuring 12 gigabytes RAM, 3072 CUDA cores, and a bandwidth of 336.5 GB/s, while the second uses a NVIDIA GTX 1080 Ti GPU featuring 11 gigabytes RAM, 3584 CUDA cores and a bandwidth of 484 GB/s. We used Python as programming language, and specifically the Numpy library for matrix multiplications, data preprocessing and segmentation, scikit-learn for training the conventional machine learning algorithms and the Keras high-level neural networks library using as backend the TensorFlow library. In order to accelerate the tensor multiplications, we used CUDA Toolkit in support with the cuDNN, which is the NVIDIA GPU-accelerated library for deep neural networks. Both workstations have 16.04 Ubuntu Linux operating system. The trained model was trained using the Adam optimizer [75] with the following hyper-parameters: learning rate = 0.005, beta_1 = 0.9, beta_2 = 0.999, epsilon = 1 × 10^−8^, decay = 0.0. Moreover, we set the minimum number of epochs to 2000; however, the training procedure terminated automatically whether the best training accuracy had improved or not, after a threshold of 100 epochs. The training epoch that achieved the lowest error rate on the validation set was saved, and its filters were used to obtain the accuracy of the model on the test set. 

Table 2 presents the obtained mean accuracy results using the deep ConvNet (deep CNN TR) described in [51] and applying transfer learning afterwards (Section 3.2) compared with the results of the deep CNN model and three base line models (random forest, Support Vector Machines, and k-Nearest Neighbors) that use hand-crafted features introduced in [47] In addition to this we designed and trained a fine-tuned deep CNN model that utilizes the batch normalization function and uses global max pooling instead of dense layers. The architecture of this model is presented in Table 3. In particular, *C* denotes the 2D convolutional layers, *BN/RL* presents the batch normalization layers, that are followed by a ReLU activation, *(G)MP* depicts the (global) max pooling layers, *Dr* presents the dropout layers, *D* denotes the dense (fully-connected) layers and, finally, *S* depicts the softmax function. Moreover, we include the results of the semi-supervised TL approach described in [52] and the human performance [49]. The Deep CNN TL model and those used for comparison in this study are evaluated in terms of classification accuracy. The selected ESC dataset is already divided into five folds, and all the models are evaluated using 5-fold cross validation. To this end, for training the Deep CNN TL model we use one of the five training folds in each split as a validation set for identifying the training epoch that yields the best model parameters when training with the remaining three folds, and we let one fold out to be used as test set in order to evaluate the model’s performance.

However, since we are interested only in identifying only a subset of ESC-50, we selected only 10 classes of as to be mapped into the seven ASoIs (Table 2). Since there some ASoIs that do not match exactly to the dataset’s ASoIs, we integrated the classes of chirping birds and crow as birds, the classes of breathing and footsteps as human, the classes of car horn and engine as mechanical. All the other classes are categorized as other. 

**Figure 9:**
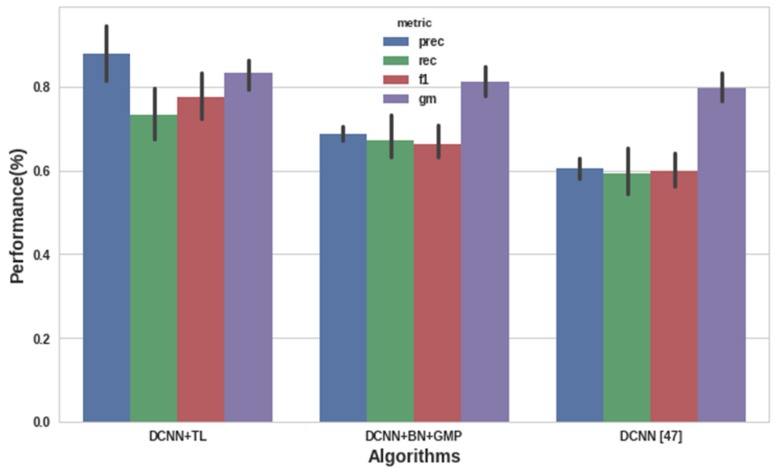
Performance results of deep CNN algorithms on detecting the seven ASoIs.

Figure 9 illustrates the results we obtained from three state-of-the-art algorithms that we trained for detecting the seven ASoIs. In particular, we retrained the Deep CNN TL, the Deep CNN BN GMP and the Deep CNN [47] having as input the ESC50 dataset. Accuracy is not included in the selected metrics due to class imbalance (i.e., the other class consists of 1600 samples which is the 80% of the whole dataset), since this metric is affected by the changes in the class distribution [76,77]. Thus, we used metrics that are not sensitive to class imbalance and can be used for selecting the most efficient algorithm on detecting small volumes of positive classes, such as the f1-score [78] and the Geometric Mean (GM) [76]. As it is presented in Figure 9, the Deep CNN TL outperformed the other two algorithms in mean precision (87.91%), recall (73.32%), f1-score (77.69%) and GM (83.32%).

**Figure 10:**
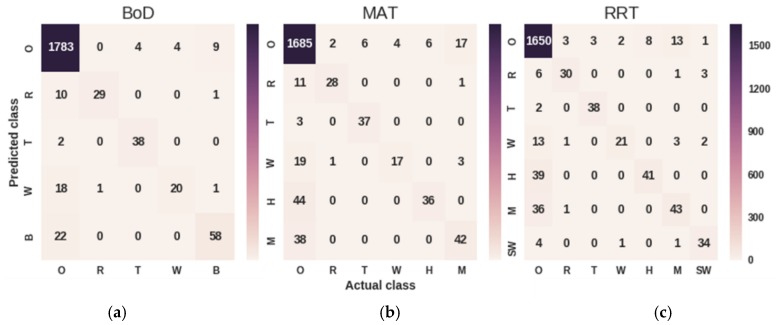
Demonstration of the results of the deep CNN TL approach per site of interest using confusion matrices: (**a**) presents the results regarding the five ASoIs of the BoD site; (**b**) presents the results regarding the six ASoIs of the MAT site; (**c**) presents the results regarding the seven ASoIs of the RRT site.

Moreover, we evaluated the Deep CNN TL model across the three sites of interest. Figure 10 presents the confusion matrices obtained by the model when trained on different ASoIs (Table 1). 

**Figure 11:**
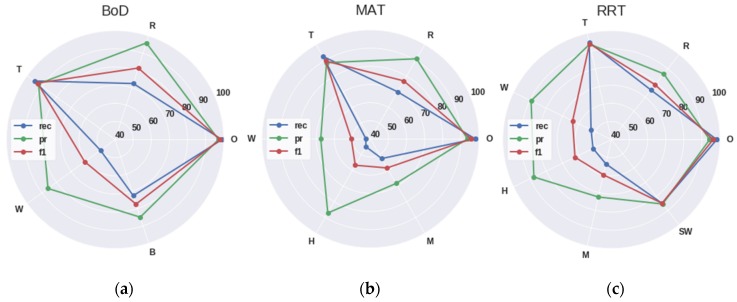
Demonstration of the results of the deep CNN TL approach per site of interest using the precision (pr), recall (rec) and f1-score (f1) metrics: (**a**) presents the results regarding the five ASoIs of the BoD site; (**b**) presents the results regarding the six ASoIs of the MAT site; (**c**) presents the results regarding the seven ASoIs of the RRT site.

It should be noted that the O symbol represents the class other, which contains audio sources that are not included in the ASoIs. As it is depicted in the confusion matrices the model struggles to distinguish audio signals produced by the wind, human and machines, while it has high precision and recall on the identifying thunderstorm sounds. As a result, the algorithm performs achieves better results (average f1-score: 0.8213 and GM: 8583) in the BoD site, since anthropogenic sounds are out of interest. The (pr), recall (rec) and f1-score (f1) metrics of each ASoI for each site of interest are demonstrated in Figure 11, using radar charts.

### 4.4. STORM Audio -Based Semsantic Rules

**Figure 12:**
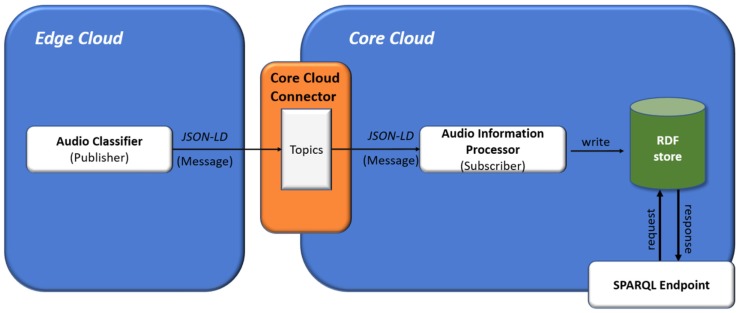
STORM audio information processing flow.

Semantic rules are used in order to apply deductive reasoning to extracted information from audio signals combined with spatiotemporal information. Figure 12 presents the entire audio information processing flow in STORM platform. In particular, the Audio Classifier component that is deployed in the Edge cloud processes real-time audio signals and extracts information regarding the source of the signal (e.g., thunderstorm), as described in the above subsection.

Afterwards, the Audio Classifier component represents the extracted information using JSON-LD format Table 4. JSON-LD [79] is a lightweight Linked Data format [80]. It is based on the JSON format, which makes it human-readable and writable, and provides a way to help JSON data interoperate semantic annotations in order to add context information and hyperlinks to the elements included in the JSON object. 

The JSON-LD files are forwarded to the interface of the Core cloud, named Core Cloud Connector (CCC) [14], which utilizes the Pub/Sub pattern via the Kafka framework [81] and makes the information available under the audio information topic. The Audio Information Processor component, which is subscribed to the relevant topic, receives real-time audio information in JSON-LD files and, after parsing them, converts them in N-Triples format (Table 4) based on the SAS-ont.

Moreover, the Audio Information Processor consists of two threads. The first one stores the produced triples into an RDF database, while the second processes the incoming information in order to generate events. An event instance is generated if the corresponding rule is active. More complex events can be formed by combining smaller rules in various ways.

Table 5 presents the audio semantic rules in Jena format [82], which are a set of RDF triples that are based on all STORM ontologies (i.e., Core, Sensor, Audio Signal and Event). For example, the rule *highWavesRRT* is activated if there exists an acoustic sensor that is located in the Roman Ruins of Tróia site, and this sensor has recorded an audio signal that has as source the class sea wave.

Finally, both the audio information and the events are publicly available through a SPARQL endpoint serving the Linked Data paradigm. SPARQL is a graph pattern matching language (RDF query language) used to retrieve and filter events or information that are stored in a Semantic Web format (e.g., N-Triples). Another feature derived from the Semantic Web is that of Linked Data that describes a method of publishing data corresponding to URIs and RDF schemas of existing ontologies capable of being interlinked with each other through the use of semantic queries. As a result, Linked Data, also called Web of Data, achieve large scale integration of data on the Web that enable developers to create cognitive applications. Advanced STORM applications, in order to be context-aware and make decisions, need the expressive power of these links and the deductive reasoning of semantic rules.

STORM Core cloud offers the produced audio events in the Linked Open Data (LOD) format. In particular, an Apache Jena tripe store [82] is hosted in the Core cloud and the Audio Information Processing component stores them in the dataset *storm-sensor-data*, which can be accessed by stakeholders, even in cases (s) he is not a STORM partner, via an endpoint produced by the Apache Jena Fuseki server [82]. 

**Figure 13:**
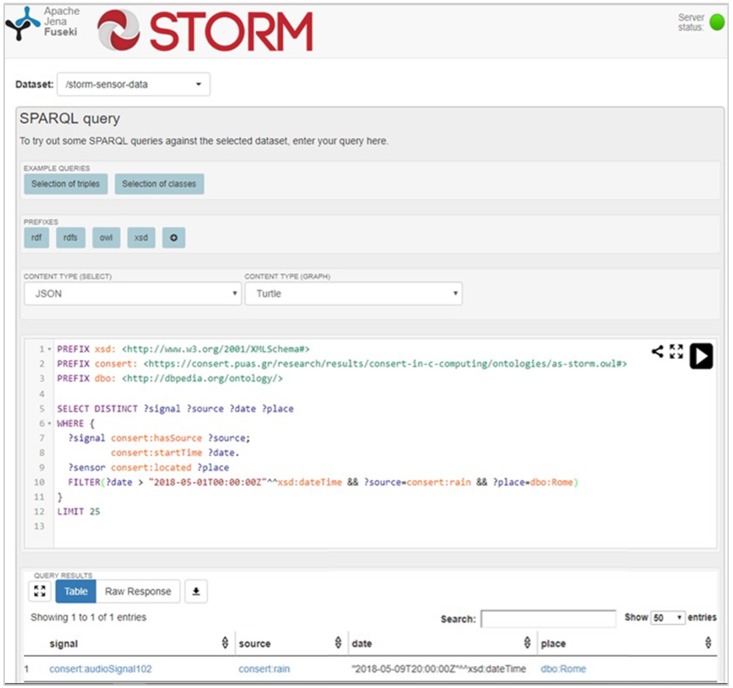
STORM Linked Open Data endpoint for SPARQL queries [84].

For example, Figure 13 illustrates a SPARQL example that retrieves the signal having as source rain and occurred in Rome (near the Baths of Diocletian site) and after the 1st of May. The current query not only makes use of the STORM-designed Audio ontology, but also takes advantage of the DBpedia Ontology [83], which is a cross-domain ontology that has been manually created based on the most commonly used infoboxes within Wikipedia. The ontology covers main 685 classes and are described by 2795 different properties. Through the use of LOD STORM structured data are interlinked with other existing datasets/ontologies (e.g., DBpedia, Geo ontology) and become more useful for researchers and stakeholders through semantic queries. Finally, the STORM LOD endpoint gives, also, its users the capability to download the retrieved data in a CSV format.

## 5. Conclusions and Future Work

In this paper we advocate that acoustic sensors can be used for monitoring and safeguarding cultural sites. The use of acoustic signals in order to detect hazardous events has many benefits compared to other methods such as the use of cameras or specialized sensors. There are quite a few cases where sound-based detection of hazardous situations can be achieved, including extreme weather phenomena such as thunderstorms, heavy rain or hail, and anthropogenic risks, such as unauthorized entry and trespassing.

To this end, we developed and employed a novel Wireless Acoustic Sensor Network that utilizes several network protocols to transmit the data to a cloud-based infrastructure. We propose a data flow framework that processes the recorded audio signals, after extracting the log-scaled mel-spectrogram, using a deep convolutional neural network that manages to surpass even the human performance. Afterwards, the extracted information is fused with spatiotemporal information to generate events that may be hazardous for the sites and their assets. Consequently, we designed the STORM Audio Signal ontology to represent the extracted audio information and process it using semantic rules. This work is part of STORM, a HORIZON 2020 funded European Project on cultural heritage; hence, we designed the sematic rules specifically for detecting hazardous events for the cultural sites participating as project partners. The results of the presented audio-based data flow are publicly available using the Linked Open Data format giving valuable insight to cultural experts and statisticians. In our ongoing work, we are planning to design a further improved event detection system by incorporating data collected by environmental sensors (e.g., humidity sensor) in order to accomplish more efficient data fusion. We also intend to transform the preprocessing and audio classification steps in order for them to be executed at the edge of the network (i.e., on the WASN). Finally, we are also planning to add interactive visualizations in order to present in a clearer way the situational picture of a hazardous event to cultural heritage stakeholders.

## Figures and Tables

**Table 1:** Audio Sources of Interest.

Class	SubClass	Symbol	RRT	BOD	MAT
Geophony	Rain	R	Yes	Yes	Yes
	Sea Waves	SW	Yes	No	No
	Thunderstorm	T	Yes	Yes	Yes
	Wind	W	Yes	Yes	Yes
Biophony	Birds	B	No	Yes	No
Anthropogenic	Human	H	Yes	No	Yes
	Mechanical	M	Yes	No	Yes

**Table 2:** Comparison of Deep CNN TR to other state-of-the-art methods.

Method Name	ESC-50
Deep CNN [47]	64.5%
Deep CNN + Batch Normalization + Global Max Pooling	73.15%
Random Forest [49]	44.3%
Support Vector Machines (SVM) [49]	39.6%
k-Nearest Neighbors (k-NN) [49]	32.2%
Semi-supervised TL [52]	79.3%
Human Performance [49]	81.3%
Deep CNN TL	81.65%

**Table 3:** Deep CNN model architecture enhanced with batch normalization layers and global max pooling.

Layer	1				2				3				4	
type	C	BN/RL	MP	Dr	C	BN/RL	MP	Dr	C	BN/RL	GMP	Dr	D	S
filter	5 × 5	n/a	4 × 4	n/a	5 × 5	n/a	2 × 2	n/a	5 × 5	n/a	n/a	n/a	128 × 50	n/a
kernel size	32	n/a	n/a	n/a	64	n/a	n/a	n/a	128	n/a	n/a	n/a	n/a	n/a
prob	n/a	n/a	n/a	0.25	n/a	n/a	n/a	0.25	n/a	n/a	n/a	0.5	n/a	n/a

**Table 4:** Example displaying the conversion of a JSON-LD file containing audio information to a N-Triples file.

Data Format	Example
JSON_LD	{ "@context": { "audio": "https://consert.puas.gr/ontologies/as-storm.owl#", "xsd": "http://www.w3.org/2001/XMLSchema#", "sensor": "https://storm.inov.pt/ontologies/storm_sensors_ontology.owl#", "audio:hasSource": { "@type": "@id" }, "audio:produces": { "@type": "@id" }, "audio:startTime": { "@type": "xsd:dateTime" }, "audio:endTime": { "@type": "xsd:dateTime" } }, "@graph": [ { "@id": "audio:sensorBOD", "@type": "sensor:AcousticSensor", "audio:produces": "audio:audioSignal101", "audio:id": "59e9a3aee74cae04002b1a33" }, { "@id": "audio:audioSignal101", "@type": "audio:audioSignal", "audio:hasSource": "audio:thunderstorm", "audio:startTime": "2018-05-09T20:00:00Z", "audio:endTime": "2018-05-09T20:00:05Z" }, { "@id": "audio:thunderstorm", "@type": "audio:audioSource" } ] }
N-Triples	@prefix audio: <http://consert.puas.gr/ontologies/as-storm.owl#> @prefix core: <http://demo-storm.eng.it/ontologies/storm_core_ontology.owl#> @prefix event: <http://demo-storm.eng.it/ontologies/storm_event_ontology.owl#> @prefix sensor: <https://storm.inov.pt/ontologies/storm_sensors_ontology.owl> @prefix rdf: < http://www.w3.org/1999/02/22-rdf-syntax-ns# > @prefix xsd: < http://www.w3.org/2001/XMLSchema > <https://consert.puas.gr/ontologies/as-storm.owl#audioSignal101> rdf:type audio:audioSignal; audio:startTime "2018-05-09T20:00:00Z"^^<http://www.w3.org/2001/XMLSchema#dateTime>; audio:endTime "2018-05-09T20:00:05Z"^^<http://www.w3.org/2001/XMLSchema#dateTime>; audio:hasSource audio:thunderstorm. <https://consert.puas.gr/ontologies/as-storm.owl#sensorBOD> rdf:type sensor:AcousticSensor; audio:id "59e9a3aee74cae04002b1a33"; audio:produces audioSignal101. <https://consert.puas.gr/ontologies/as-storm.owl#thunderstorm> rdf:type audio:audioSource.

**Table 5:** List of STORM audio semantic rules in Jena format.

**List of Prefixes**	
audio: <http://consert.puas.gr/ontologies/as-storm.owl#> core: <http://demo-storm.eng.it/ontologies/storm_core_ontology.owl#> event: <http://demo-storm.eng.it/ontologies/storm_event_ontology.owl#> sensor: <https://storm.inov.pt/ontologies/storm_sensors_ontology.owl> rdf: < http://www.w3.org/1999/02/22-rdf-syntax-ns# >	
** Rule Name**	** Description in Jena rules**
*strongWindsBOD*	(?s rdf:type sensor:AcousticSensor) (?a rdf:type audio:AudioSignal) (?s sensor:hasData ?a) (?s core:placed core:BathsOfDiocletian) (?a audio:hasSource audio:wind) -> (?a core:initiate ?e) (?e event:hasCategory event:strongWinds)
*strongWindsΜAΤ*	(?s rdf:type sensor:AcousticSensor) (?a rdf:type audio:AudioSignal) (?s sensor:hasData ?a) (?s core:placed core:MellorSite) (?a audio:hasSource audio:wind) -> (?a core:initiate ?e) (?e event:hasCategory event:strongWinds)
*strongWindsRRT*	(?s rdf:type sensor:AcousticSensor) (?a rdf:type audio:AudioSignal) (?s sensor:hasData ?a) (?s core:placed core:RomanRuinsOfTroia) (?a audio:hasSource audio:wind) -> (?a core:initiate ?e) (?e event:hasCategory event:strongWinds)
*highWavesRRT*	(?s rdf:type sensor:AcousticSensor) (?a rdf:type audio:AudioSignal) (?s sensor:hasData ?a) (?s core:placed core:RomanRuinsOfTroia) (?a audio:hasSource audio:seawaves) -> (?a core:initiate ?e) (?e event:hasCategory event:highWaves)
*thunderStormBOD*	(?s rdf:type sensor:AcousticSensor) (?a rdf:type audio:AudioSignal) (?s sensor:hasData ?a) (?s core:placed core:BathsOfDiocletian) (?a audio:hasSource audio:thunderstorm) -> (?a core:initiate ?e) (?e event:hasCategory event:thunderStorm)
*thunderStormMAT*	(?s rdf:type sensor:AcousticSensor) (?a rdf:type audio:AudioSignal) (?s sensor:hasData ?a) (?s core:placed core:MellorSite) (?a audio:hasSource audio:thunderstorm) -> (?a core:initiate ?e) (?e event:hasCategory event:thunderStorm)
*thunderStormRRT*	(?s rdf:type sensor:AcousticSensor) (?a rdf:type audio:AudioSignal) (?s sensor:hasData ?a) (?s core:placed core:RomanRuinsOfTroia) (?a audio:hasSource audio:thunderstorm) -> (?a core:initiate ?e) (?e event:hasCategory event:thunderStorm)
*intenseRainfallBOD*	(?s rdf:type sensor:AcousticSensor) (?a rdf:type audio:AudioSignal) (?s sensor:hasData ?a) (?s core:placed core:BathsOfDiocletian) (?a audio:hasSource audio:rain) -> (?a core:initiate ?e) (?e event:hasCategory event:intenseRainfall)
*intenseRainfallMAT*	(?s rdf:type sensor:AcousticSensor) (?a rdf:type audio:AudioSignal) (?s sensor:hasData ?a) (?s core:placed core:MellorSite) (?a audio:hasSource audio:rain) -> (?a core:initiate ?e) (?e event:hasCategory event:intenseRainfall)
*intenseRainfallRRT*	(?s rdf:type sensor:AcousticSensor) (?a rdf:type audio:AudioSignal) (?s sensor:hasData ?a) (?s core:placed core:RomanRuinsOfTroia) (?a audio:hasSource audio:rain) -> (?a core:initiate ?e) (?e event:hasCategory event:intenseRainfall)
*animalStampedeBOD*	(?s rdf:type sensor:AcousticSensor) (?a rdf:type audio:AudioSignal) (?s sensor:hasData ?a) (?s core:placed core:BathsOfDiocletian) (?a audio:hasSource audio:bird) -> (?a core:initiate ?e) (?e event:hasCategory event:animalStampede)
*possibleVandalismMAT*	(?s rdf:type sensor:AcousticSensor) (?a rdf:type audio:AudioSignal) (?s sensor:hasData ?a) (?h rdf:type audio:Anthropogenic) (?s core:placed core:MellorSite) (?a audio:hasSource ?h) (?a audio:hour ?hst) lessThan(?hst, 7) greaterThan(?hst, 22) -> (?a core:initiate ?e) (?e event:hasCategory event:vandalism)]
*possibleVandalismRRT*	(?s rdf:type sensor:AcousticSensor) (?a rdf:type audio:AudioSignal) (?s sensor:hasData ?a) (?h rdf:type audio:Anthropogenic) (?s core:placed core:RomanRuinsOfTroia) (?a audio:hasSource ?h) -> (?a core:initiate ?e) (?e event:hasCategory event:vandalism)
